# Recursive Minimum Complex Kernel Risk-Sensitive Loss Algorithm

**DOI:** 10.3390/e20120902

**Published:** 2018-11-25

**Authors:** Guobing Qian, Dan Luo, Shiyuan Wang

**Affiliations:** 1 College of Electronic and Information Engineering, Brain-inspired Computing & Intelligent Control of Chongqing Key Laboratory, Chongqing Key Laboratory of Nonlinear Circuits and Intelligent Information Processing, Southwest University, Chongqing 400715, China; 2 School of Mathematics and Statistics, Southwest University, Chongqing 400715, China

**Keywords:** complex, kernel risk-sensitive loss, recursive, stability, EMSE

## Abstract

The maximum complex correntropy criterion (MCCC) has been extended to complex domain for dealing with complex-valued data in the presence of impulsive noise. Compared with the correntropy based loss, a kernel risk-sensitive loss (KRSL) defined in kernel space has demonstrated a superior performance surface in the complex domain. However, there is no report regarding the recursive KRSL algorithm in the complex domain. Therefore, in this paper we propose a recursive complex KRSL algorithm called the recursive minimum complex kernel risk-sensitive loss (RMCKRSL). In addition, we analyze its stability and obtain the theoretical value of the excess mean square error (EMSE), which are both supported by simulations. Simulation results verify that the proposed RMCKRSL out-performs the MCCC, generalized MCCC (GMCCC), and traditional recursive least squares (RLS).

## 1. Introduction

As many noises are non-Gaussian distributed in practice, the performance of traditional second-order statistics-based similarity measures may deteriorate dramatically [1,2]. To efficiently handle the non-Gaussian noise, a higher order statistic called correntropy [3,4,5,6] was proposed. The correntropy is a nonlinear and local similarity measure widely used in adaptive filters [7,8,9,10,11,12,13,14,15], and usually employs a Gaussian function as the kernel function thanks to flexible and positive definiteness. However, the Gaussian kernel is not always the best choice [16]. Hence, Chen et al. proposed the generalized maximum correntropy criterion (GMCC) algorithm [16,17] using a generalized Gaussian density function as the kernel. Compared with traditional maximum correntropy criterion (MCC), the GMCC behaves better when the shape parameter is properly selected. In addition, the MCC can be regarded as a special case of GMCC. Considering that the error performance surface of correntropic loss is highly non-convex, Chen et al. proposed another algorithm named the minimum kernel risk-sensitive loss (MKRSL), which is defined in kernel space but also inherits the original form of risk-sensitive loss (RSL) [18,19]. The performance surface of the kernel risk-sensitive loss (KRSL) is more efficient than the MCC, resulting in a faster convergence speed and a higher accuracy. Furthermore, KRSL is also not sensitive to outliers.

Generally, adaptive filter has been mainly focused on the real domain and cannot be used to deal with complex-valued data directly. Recently, the complex domain adaptive filter has drawn more attention. Guimaraes et al. proposed the maximum complex correntropy criterion (MCCC) [20,21] and provided a probabilistic interpretation [20]. MCCC shows an obvious advantage over the least absolute deviation (LAD) [22], complex least mean square (CLMS) [23], and recursive least squares (RLS) algorithms [24]. The stability analysis and the theoretical EMSE of the MCCC have been derived [25]. The MCCC has been extended to the generalized case [26]. The generalized MCCC (GMCCC) algorithm employs a complex generalized Gaussian density as a kernel and offers a desirable performance for handling the complex-valued data. In addition, a gradient-based complex kernel risk-sensitive loss (CKRSL) defined in kernel space has shown a superior performance [27]. Until now, there has been no report about the recursive complex KRSL (CKRSL) algorithm. Therefore, in this paper we first propose a recursive minimum CKRSL (RMCKRSL) algorithm. Then, we analyze the stability and calculate the theoretical value of the EMSE. Simulations show that the RMCKRSL is better than the MCCC, GMCCC, and traditional RLS. Simultaneously, the correctness of the theoretical analysis is also demonstrated by simulations.

The remaining parts of this paper are organized as follows: In Section 2, we provide the loss function of the CKRSL and propose the recursive MCKRSL algorithm. In Section 3 we analyze the stability and obtain the theoretical value of the EMSE for the proposed algorithm. In Section 4, simulations are performed to verify the superior convergence of the RMCKRSL algorithm and the correctness of the theoretical analysis. Finally, in Section 5 we draw a conclusion. 

## 2. Fixed Point Algorithm under Minimizing Complex Kernel Risk-Sensitive Loss

### 2.1. Complex Kernel Risk-Sensitive Loss

Supposing there are two complex variables $C_{1}=X_{1}+jY_{1}$ and $C_{2}=X_{2}+jY_{2}$, the complex kernel risk-sensitive loss (CKRSL) is defined as [27]:

(1) $$L_{\lambda }^{c}\left(C_{1},C_{2}\right)=\frac{1}{\lambda }E\left[\exp\left[\lambda \left(1-\kappa_{\sigma }^{c}\left(C_{1}-C_{2}\right)\right)\right]\right]\;$$

where $X_{1}$, $X_{2}$, $Y_{1}$ and $Y_{2}$ are real variables, λ is the risk-sensitive parameter, and $\kappa_{\sigma }^{c}\left(C_{1}-C_{2}\right)$ is the kernel function.

This paper employs a Gaussian kernel which is expressed as:

(2) $$\kappa_{\sigma }^{c}\left(C_{1}-C_{2}\right)=\exp\left(-\frac{\left(C_{1}-C_{2}\right){\left(C_{1}-C_{2}\right)}^{*}}{2\sigma^{2}}\right)\;$$

where σ is the kernel width.

### 2.2. Recursive Minimum Complex Kernel Risk-Sensitive Loss (RMCKRSL)

#### 2.2.1. Cost Function 

We define the cost function of MCKRSL as:

(3) $$\begin{array}{ll} J_{MCKRSL}^{} & =E\left[L_{\lambda }^{c}\left(e\left(k\right)\right)\right]\\ & =\frac{1}{\lambda }E\left[\exp\left[\lambda \left(1-\kappa_{\sigma }^{c}\left(e\left(k\right)\right)\right)\right]\right] \end{array}$$

where

(4) $$e\left(k\right)=d\left(k\right)-w^{H}x\left(k\right)\;$$

denotes the error at the $k^{th}$ iteration, d(k) represents the expected response at the $k^{th}$ iteration, $w=\left[\begin{array}{cccc} w_{1} & w_{2} & \cdots & w_{m} \end{array}\right]$ denotes the estimated weight vector, m is the length of adaptive filter, and $x\left(k\right)=[x\left(k\right){\begin{array}{ccc} x\left(k-1\right) & \cdots & x\left(k-m+1\right) \end{array}]}^{T}$ is the input vector, ${\left(\cdot \right)}^{H}$ and ${\left(\cdot \right)}^{T}$ denote the conjugate transpose and transpose, respectively. 

#### 2.2.2. Recursive Solution

Using the Wirtinger Calculus [28,29], the gradient of $J_{MCKRSL}^{}$ with respect to $w^{*}$ is derived:

(5) $$\frac{\partial J_{MCKRSL}^{}}{\partial w^{*}}=E\left[L_{\lambda }^{c}\left(e\right)\times \left(-d^{*}x+xx^{H}w\right)\right]\;$$

By making $\frac{\partial J_{MCKRSL}^{}}{\partial w^{*}}=0$, we obtain the optimal solution

(6) $$w=R_{}^{-1}p\;$$

where

(7) $$R=E\left\{h\left(e\right)xx^{H}\right\}\;$$

(8) $$p=E\left\{h\left(e\right)d^{*}x\right\}$$

(9) $$h\left[e\right]=\exp\left[\lambda \left(1-\kappa_{\sigma }^{c}\left(e\right)\right)\right]\kappa_{\sigma }^{c}\left(e\right)\;$$

It is noted that Equation (6) is actually a fixed point solution because R and p depend on w. In practice, R and p are usually estimated as follows when the samples are finite:

(10) $$\hat{R}=\frac{1}{N}\sum_{k=1}^{N}h\left[e\left(k\right)\right]x\left(k\right)x^{H}\left(k\right)\;$$

(11) $$\hat{p}=\frac{1}{N}\sum_{k=1}^{N}h\left[e\left(k\right)\right]d^{*}\left(k\right)x\left(k\right)\;$$

Hence, $\hat{R}$, $\hat{p}$ and w are updated as follows:

(12) $$\begin{array}{c} {\hat{R}}_{k}={\hat{R}}_{k-1}+h\left[e\left(k\right)\right]x\left(k\right)x^{H}\left(k\right)\\ {\hat{p}}_{k}={\hat{p}}_{k-1}+h\left[e\left(k\right)\right]d^{*}\left(k\right)x\left(k\right)\\ w\left(k\right)={\hat{R}}_{k}{}^{-1}\;{\hat{p}}_{k} \end{array}$$

Using the matrix inversion lemma [30], we may rewrite ${\hat{R}}_{k}^{-1}$ in Equation (12) as:

(13) $${\hat{R}}_{k}^{-1}={\hat{R}}_{k-1}^{-1}-{\hat{R}}_{k-1}^{-1}x\left(k\right){\left(h^{-1}\left(e\left(k\right)\right)+x^{H}\left(k\right){\hat{R}}_{k-1}^{-1}x\left(k\right)\right)}^{-1}\times x^{H}\left(k\right){\hat{R}}_{k-1}^{-1}\;$$

(14) $$b\left(k\right)={\hat{R}}_{k-1}^{-1}x\left(k\right){\left(h^{-1}\left(e\left(k\right)\right)+x^{H}\left(k\right){\hat{R}}_{k-1}^{-1}x\left(k\right)\right)}^{-1}\;$$

(15) $$\left(h^{-1}\left(e\left(k\right)\right)+x^{H}\left(k\right){\hat{R}}_{k-1}^{-1}x\left(k\right)\right)b\left(k\right)={\hat{R}}_{k-1}^{-1}x\left(k\right)\;$$

and

(16) $$\begin{array}{ll} b\left(k\right) & =h\left(e\left(k\right)\right){\hat{R}}_{k-1}^{-1}x\left(k\right)\\ & \;-b\left(k\right)h\left(e\left(k\right)\right)x^{H}\left(k\right){\hat{R}}_{k-1}^{-1}x\left(k\right)\\ & =h\left(e\left(k\right)\right)\left({\hat{R}}_{k-1}^{-1}-b\left(k\right)x^{H}\left(k\right){\hat{R}}_{k-1}^{-1}\right)x\left(k\right)\\ & =h\left(e\left(k\right)\right){\hat{R}}_{k}^{-1}x\left(k\right) \end{array}$$

After some algebraic manipulations, we may derive the recursive form of w(k) as follows:

(17) $$\begin{array}{ll} w\left(k\right) & ={\hat{R}}_{k}{}^{-1}\;\left\{h\left(e\left(k\right)\right)d^{*}\left(k\right)x\left(k\right)+{\hat{p}}_{k-1}\right\}\\ & =w\left(k-1\right)+b\left(k\right)e^{*}\left(k\right) \end{array}$$

Finally, Algorithm 1 summarizes the recursive MCKRSL (RMCKRSL) algorithm.
**Algorithm 1:** RMCKRSL.**Input:**σ, λ, d(k), x(k) 1. Initializations: δ=0.0001, $\;p_{0}=0$, w(0)=0, $R_{0}=\delta I$, $R_{0}^{-1}=\delta^{-1}I$ 2. While $\left\{\begin{array}{cc} x\left(k\right) & d\left(k\right) \end{array}\right\}$ available, do 3. $e\left(k\right)=d\left(k\right)-w^{H}\left(k\right)x\left(k\right)$ 4. $\kappa_{\sigma }^{c}\left(e\left(k\right)\right)=\exp\left(-{\left|e\left(k\right)\right|}^{2}/2\sigma^{2}\right)$ 5. $h\left[e\left(k\right)\right]=\exp\left[\lambda \left(1-\kappa_{\sigma }^{c}\left(e\left(k\right)\right)\right)\right]\kappa_{\sigma }^{c}\left(e\left(k\right)\right)$ 6. $b\left(k\right)={\hat{R}}_{k-1}^{-1}x\left(k\right){\left(h^{-1}\left(e\left(k\right)\right)+x^{H}\left(k\right){\hat{R}}_{k-1}^{-1}x\left(k\right)\right)}^{-1}$ 7. $w\left(k\right)=w\left(k-1\right)+b\left(k\right)e^{*}\left(k\right)$ 8. ${\hat{R}}_{k}^{-1}={\hat{R}}_{k-1}^{-1}-{\hat{R}}_{k-1}^{-1}x\left(k\right){\left(h^{-1}\left(e\left(k\right)\right)+x^{H}\left(k\right){\hat{R}}_{k-1}^{-1}x\left(k\right)\right)}^{-1}\times x^{H}\left(k\right){\hat{R}}_{k-1}^{-1}$ 9. End while10. ${\hat{w}}_{0}=w\left(k\right)$
Output: Estimated filter weight ${\hat{w}}_{0}$

## 3. Convergence Analysis

### 3.1. Stability Analysis

Supposing the desired signal is as follows:

(18) $$d\left(k\right)=w_{0}^{H}x\left(k\right)+v\left(k\right)\;$$

we rewrite the error as:

(19) $$\begin{array}{ll} e\left(k\right) & ={\tilde{w}}^{H}\left(k-1\right)x\left(k\right)+v\left(k\right)\\ & =e_{a}\left(k\right)+v\left(k\right) \end{array}$$

where $w_{0}^{}$ is the system parameter to be estimated, $\tilde{w}\left(k-1\right)=w_{0}-w\left(k-1\right)$, v(k) represents the noise at discrete time k, and $e\left(k\right)={\tilde{w}}^{H}\left(k-1\right)x\left(k\right)$.

Furthermore, we rewrite w(k) as:

(20) $$\begin{array}{ll} w\left(k\right) & =w\left(k-1\right)+h\left(e\left(k\right)\right)e^{*}\left(k\right){\hat{R}}_{k}^{-1}x\left(k\right)\\ & \approx w\left(k-1\right)+\frac{a_{0}}{k}f\left(e\left(k\right)\right){\bar{R}}_{}^{-1}x\left(k\right) \end{array}$$

where $f\left(e\left(k\right)\right)=h\left(e\left(k\right)\right)e^{*}\left(k\right)$, $\bar{R}\;=E\left\{xx^{H}\right\}$, $a_{0}=$$1/E\left[\exp\left[\lambda \left(1-\kappa_{\sigma }^{c}\left(v\right)\right)\right]\kappa_{\sigma }^{c}\left(v\right)\right]$, v represents the noise, and the second line is approximately obtained by using the following:

(21) $$\begin{array}{ll} {\hat{R}}_{k}^{-1} & ={\left[\sum_{l=1}^{k}h\left[e\left(l\right)\right]x\left(l\right)x^{H}\left(l\right)\right]}^{-1}\\ & ={\left[k\left[\frac{1}{k}\sum_{l=1}^{k}h\left[e\left(l\right)\right]x\left(l\right)x^{H}\left(l\right)\right]\right]}^{-1}\overset{}{\approx }\frac{a_{0}}{k}{\bar{R}}^{-1} \end{array}$$

**Remark** **1.** *(1) We can approximate the second line of Equation (20) when*${\left|e_{a}\left(l\right)\right|}^{2}$*is small enough, where*$e_{a}\left(l\right)={\left(w_{0}-w\left(l-1\right)\right)}^{H}x\left(l\right)$.

*(2) According to Equation (20), the RMCKRSL can be approximately viewed as a gradient descend method with variable steps a_*0*_/k.*

*(3) We can estimate* $\bar{R}\;$ *by* $\frac{1}{N}\sum_{l=1}^{N}x\left(l\right)x^{H}\left(l\right)$ *, where* N *is the number of samples.*

By multiplying ${\bar{R}}_{}^{1/2}$ on both sides of Equation (20), we obtain the following:

(22) $${\bar{R}}_{}^{1/2}\tilde{w}\left(k\right)={\bar{R}}_{}^{1/2}\tilde{w}\left(k-1\right)-\frac{a_{0}}{k}f\left(e\left(k\right)\right){\bar{R}}_{}^{-1/2}x\left(k\right)$$

where $f\left(e\left(k\right)\right)=\exp\left[\lambda \left(1-\kappa_{\sigma }^{c}\left(e\left(k\right)\right)\right)\right]\kappa_{\sigma }^{c}\left(e\left(k\right)\right)e^{*}\left(k\right)$.

Therefore,

(23) E{‖R¯1/2w˜(k)‖2}=E{‖R¯1/2w˜(k−1)‖2}−2a0kE{Re[ea(k)f(e(k))]} +a02k2E{‖Im‖F2|f(e(k))|2}

where ${\left\|\cdot \right\|}_{F}^{}$ represents the Frobenius norm, Re(⋅) is the real part, and $I_{m}$ denotes the m×m identity matrix.

Then, we can determine that if

(24) k≥m a0E{|f(e(k))|2}2E{Re[ea(k)f(e(k))]} 

the sequence $E\left\{{\left\|\tilde{w}\left(k\right)\right\|}^{2}\right\}$ is decreasing and the algorithm will converge. 

### 3.2. Excess Mean Square Error

Let S(k) be the excess mean square error (EMSE) and defined as:

(25) $$S\left(k\right)=E\left[{\left|e_{a}\left(k\right)\right|}^{2}\right]$$

To derive the theoretical value of S(k), we adopt some commonly used assumptions [8,27,31]:

(A1) v(k) is zero-mean and independently identically distributed (IID); $e_{a}\left(k\right)$ is independent of v(k) and also zero-mean;

(A2) x(k) is independent of v(k), circular and stationary.

Thus, taking (23) and (25) into consideration, we obtain the following:

(26) S(k+1)=S(k)−2a0kE{Re[ea(k)f(e(k))]}+ma02k2E{|f(e(k))|2}

Similar to [27], we can obtain the following:

(27) $$\begin{array}{l} E\left\{{\left|f\left(e\left(k\right)\right)\right|}^{2}\right\}=E\left\{\exp\left[2\lambda \left(1-\kappa_{\sigma }^{c}\left(v\right)\right)\right]\kappa_{\sigma /\sqrt{2}}^{c}\left(v\right){\left|v\right|}^{2}\right\}\\ +S\left(k\right)\;\times E\;\left\{\exp\left[-2\lambda \left(\kappa_{\sigma }^{c}\left(v\right)-1\right)\right]\kappa_{\sigma /\sqrt{2}}^{c}\left(v\right)R_{1}\right\} \end{array}$$

(28) E{Re[ea(k)f(e(k))]}=S(k)E{exp[λ(1−κσc(v))]R2} 

where

(29) $$\begin{array}{ll} R_{1} & =[1-3\frac{{\left|v\right|}^{2}}{\sigma^{2}}+\frac{{\left|v\right|}^{4}}{\sigma^{4}}+3\lambda \frac{{\left|v\right|}^{2}}{\sigma^{2}}\kappa_{\sigma }^{c}\left(v\right)\\ & \;-5\lambda \frac{{\left|v\right|}^{4}}{2\sigma^{4}}\kappa_{\sigma }^{c}\left(v\right)+\frac{{\left|v\right|}^{4}}{\sigma^{4}}\lambda^{2}\kappa_{\sigma /\sqrt{2}}^{c}\left(v\right)] \end{array}$$

(30) $$R_{2}=\kappa_{\sigma }^{c}\left(v\right)\left(1+\frac{1}{2\sigma^{2}}\left(\lambda \kappa_{\sigma }^{c}\left(v\right)-1\right){\left|v\right|}^{2}\right)\;$$

Thus,

(31) $$S\left(k+1\right)=S\left(k\right)-\frac{2a_{0}a_{1}}{k}S\left(k\right)+\frac{a_{0}{}^{2}a_{2}}{k^{2}}S\left(k\right)+\frac{a_{0}{}^{2}a_{3}}{k^{2}}\;$$

where $a_{1}=E\left\{\exp\left[\lambda \left(1-\kappa_{\sigma }^{c}\left(v\right)\right)\right]R_{2}\right\}$, $a_{2}=mE\;\left\{\exp\left[-2\lambda \left(\kappa_{\sigma }^{c}\left(v\right)-1\right)\right]\kappa_{\sigma /\sqrt{2}}^{c}\left(v\right)R_{1}\right\}$, $a_{3}=mE\left\{\exp\left[2\lambda \left(1-\kappa_{\sigma }^{c}\left(v\right)\right)\right]\kappa_{\sigma /\sqrt{2}}^{c}\left(v\right){\left|v\right|}^{2}\right\}$.

It can be seen from Equation (31), that *S*(*k*) is the solution to a first-order difference equation. Thus, we derive that:

(32) $$S\left(k\right)=S_{h}\left(k\right)+S_{p}\left(k\right)\;$$

where $S_{h}\left(k\right)=c_{1}\lambda \left(k-1\right)\lambda \left(k-2\right)\cdots \lambda \left(1\right)$ is the homogeneous solution with $\lambda \left(k-1\right)=1-2a_{0}a_{1}/\left(k-1\right)+a_{0}{}^{2}a_{2}/{\left(k-1\right)}^{2}$, and $S_{p}\left(k\right)$ is the particular solution where:

(33) $$S_{p}\left(k\right)=\left(\frac{a_{0}a_{3}}{k}c_{2}\right)/\left(2a_{1}-\frac{a_{0}a_{2}}{k}c_{2}\right)\;$$

and $c_{2}\approx \frac{2a_{0}a_{1}}{2a_{0}a_{1}-\left[k/\left(k+1\right)\right]}$.

**Remark** **2.** *The theoretical value of*S(k)*in Equation (32) is reliable only when*${\left|e_{a}\right|}^{2}$*is small enough and*k*is large.*$c_{1}$ can be obtained by using the initial value of the EMSE. However, it is not necessary to calculate $c_{1}$ *in general, because* $S_{h}\left(k\right)\ll S_{p}\left(k\right)$ *when* k *is large. Thus,* $S\left(k\right)\approx S_{p}\left(k\right)$.

## 4. Simulation

In this section, two examples are used to illustrate the superior performance of the RMCKRSL i.e., system identification and nonlinear prediction. We obtained the simulation results by averaging 1000 Monte Carlo trials.

### 4.1. Example 1

We chose the length of the filter as five where the weight vector $w_{0}=\left[\begin{array}{cccc} w_{1} & w_{2} & \cdots & w_{5} \end{array}\right]$ is generated randomly, where $w_{i}=w_{Ri}+jw_{Ii}$, with $w_{Ri}\in N\left(0,\;0.1\right)$ and $w_{Ii}\in N\left(0,\;0.1\right)$ being the real and imaginary parts of $w_{i}$, and $N\left(\mu ,\;\sigma^{2}\right)$ denoting the Gaussian distribution with μ and $\sigma^{2}$ being the mean and variance, respectively. The input signal $x=x_{R}+jx_{I}$ is also generated randomly, where $x_{R},\;x_{I}\in N\left(0,\;1\right)$. An additive complex noise, $v=v_{R}+jv_{I}$, with $v_{R}$ and $v_{I}$ being the real and imaginary parts, is considered in the simulations. 

**Figure 1:**
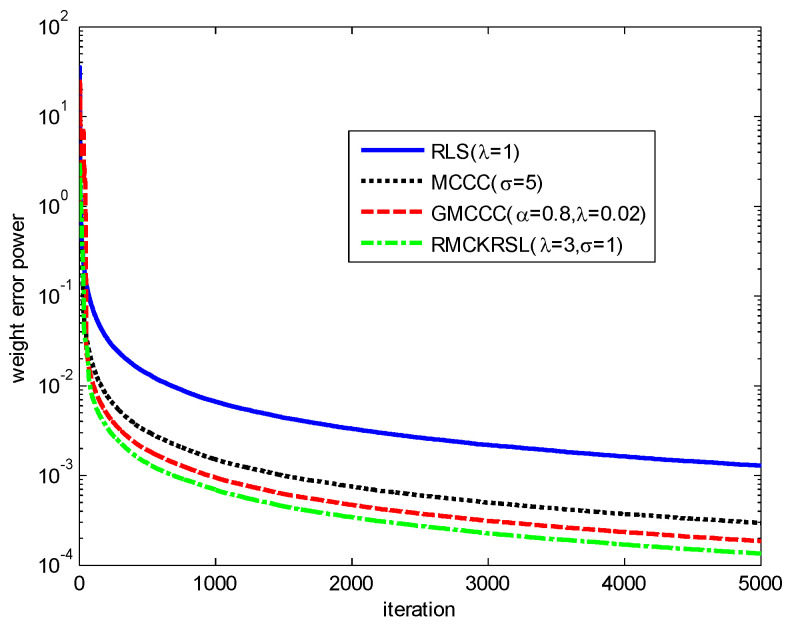
Learning curves of different algorithms.

First, we verify the superiority of the RMCKRSL in the presence of contaminated Gaussian noise [17,19], i.e., $v\left(k\right)=\left(1-c\left(k\right)\right)v_{1}\left(k\right)+c\left(k\right)v_{2}\left(k\right)$, where $v_{1}\left(k\right)=v_{1R}\left(k\right)+jv_{1I}\left(k\right)$, with $v_{1R},\;v_{1I}\in$ N(0, 0.1), $v_{2}\left(k\right)=v_{2R}\left(k\right)+jv_{2I}\left(k\right)$ with $v_{2R},\;v_{2I}\in N\left(0,\;20\right)$ represents an outlier (or impulsive disturbances), P(c(k)=1)=0.06 is the occurrence probability of impulsive disturbances, P(c(k)=0)=0.94. To ensure a fair comparison, all the algorithms use the recursive iteration to search the optimal solution. The parameters for different algorithms are chosen experimentally to guarantee the desirable solution. The performances of different algorithms on the basis of weight error power ${\left\|w_{0}-w\left(k\right)\right\|}^{2}$ are shown in Figure 1. It is clear that compared with the MCCC, GMCCC and traditional RLS, RMCKRSL has the best filtering performance.

**Figure 2:**
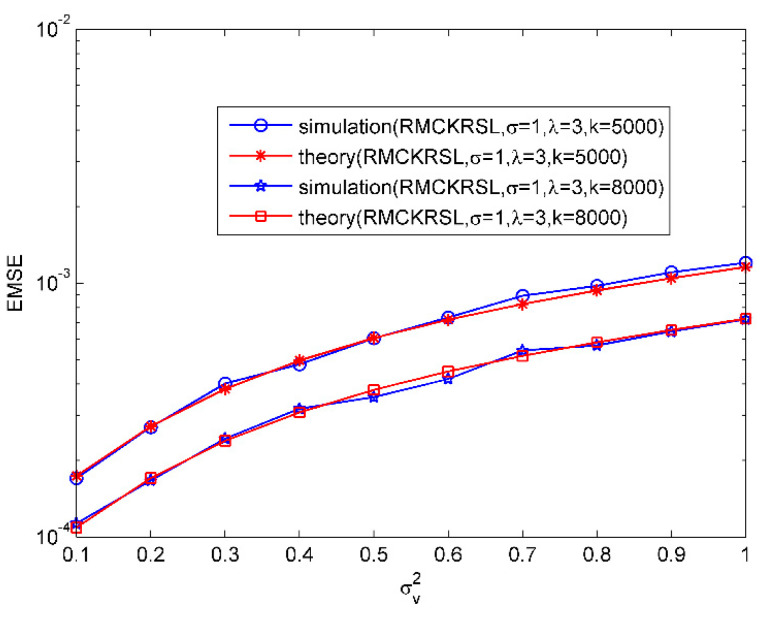
EMSE as a function of noise variances.

Then, the validity of the theoretical EMSEs for MCKRSL is demonstrated. The noise model is also a contaminated Gaussian model, where $v_{1R},\;v_{1I}\in N\left(0,\;\sigma_{v}^{2}/2\right)$, $v_{2R},\;v_{2I}\in N\left(0,\;20\right)$, P(c(k)=1)=0.06 and P(c(k)=0)=0.94. Figure 2 compares the values of the theoretical EMSEs and simulated ones under variations of $\sigma_{v}^{2}$. Obviously there is a good match between the theoretical EMSEs and the simulated ones. In addition, it has been shown that the value of EMSE becomes bigger with the increase of noise variance.

**Figure 3:**
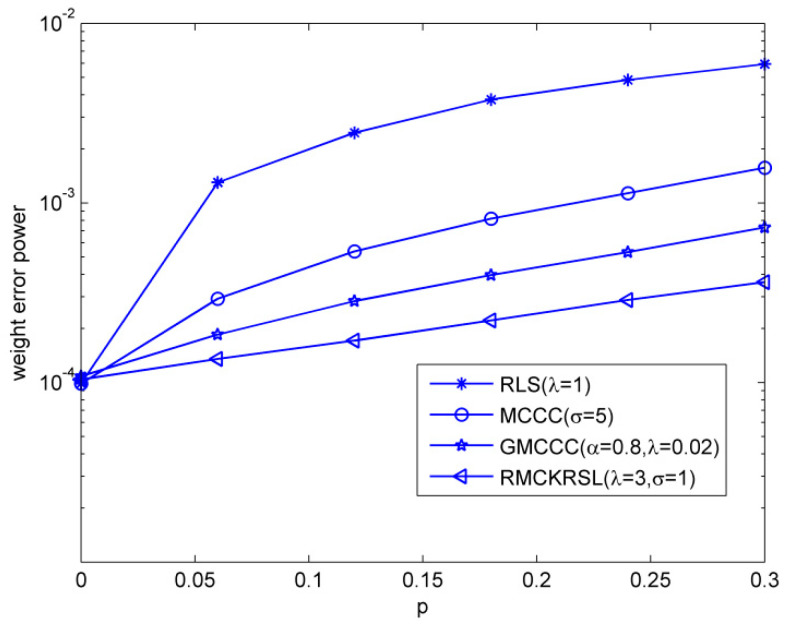
Influence of the probability of outliers.

**Figure 4:**
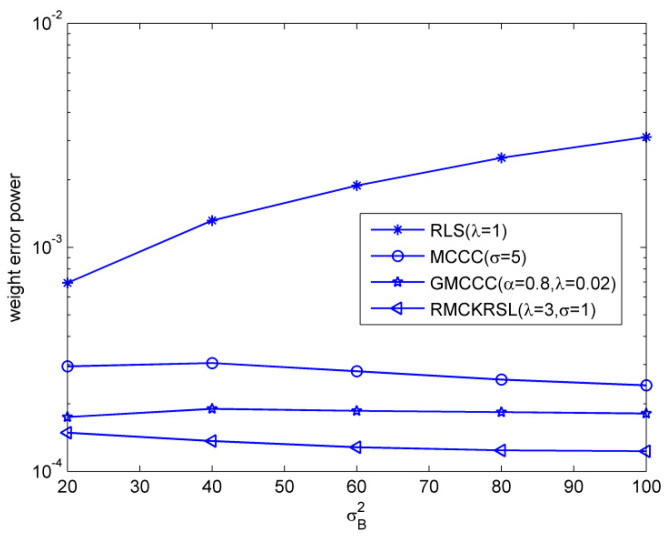
Influence of the variance of outliers.

Next, we tested the influence of the outlier on the performance of the RMCKRSL algorithm. The noise model is also a contaminated Gaussian noise, where $v_{1R},\;v_{1I}\in N\left(0,\;0.1\right)$, $v_{2R},\;v_{2I}\in N\left(0,\;\sigma_{B}^{2}/2\right)$, P(c(k)=1)=p and P(c(k)=0)=1−p. Figure 3 compares the performances of different algorithms under different probability of outlier values (p), where the sample size is 5000 and the variance of the outlier is $\sigma_{B}^{2}=40$. One can observe that the proposed RMCKRSL algorithm is robust to the probability of an outlier and has better performance than the MCCC, GMCCC and RLS. Figure 4 depicts the performances of different algorithms under different variance of outlier ($\sigma_{B}^{2}$) values, where the sample size is also 5000 and the probability of an outlier is p=0.06. It can be observed that the proposed RMCKRSL algorithm is also robust to the variance of an outlier and has better performance than other algorithms.

**Figure 5:**
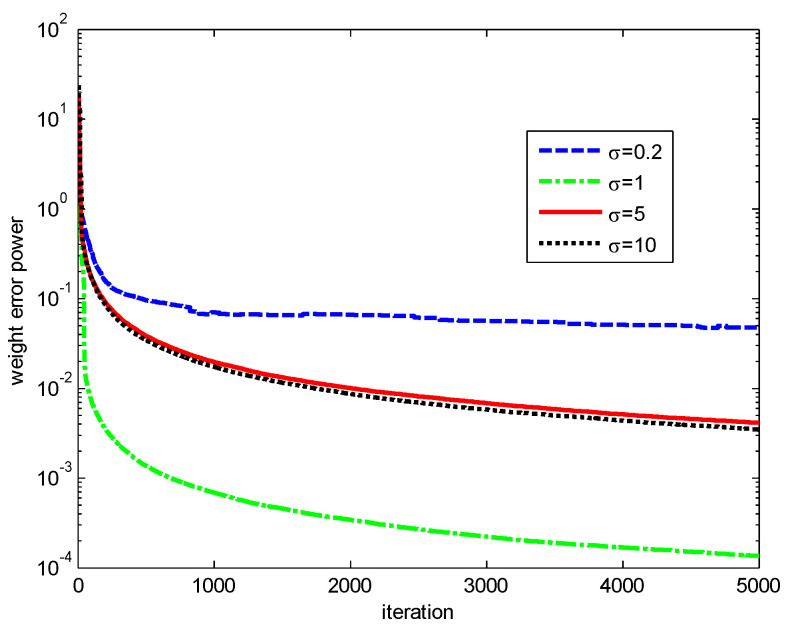
Influence of the σ (λ=3).

**Figure 6:**
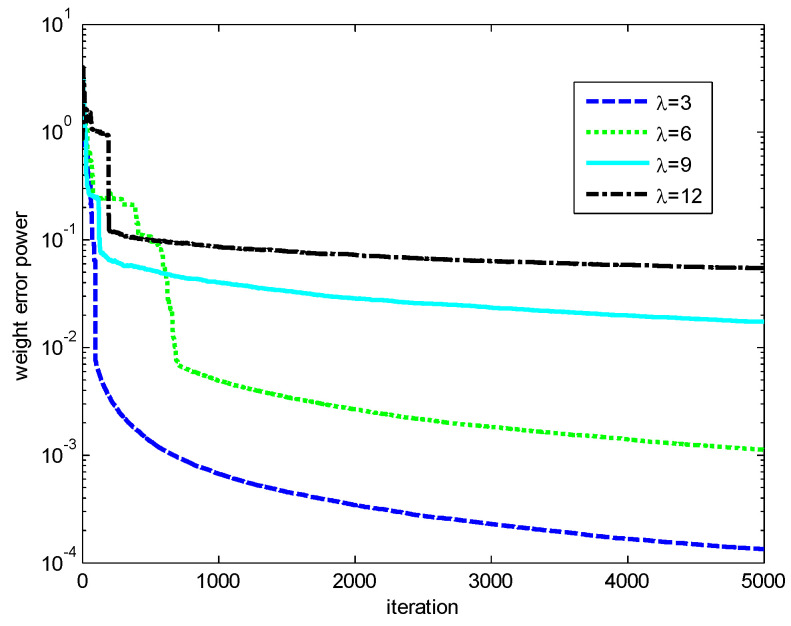
Influence of the λ (σ=1).

Finally, the influences of the kernel width σ and risk-sensitive parameter λ on the performance of the RMCKRSL are investigated. The noise model is also a contaminated Gaussian noise, where $v_{1R},\;v_{1I}\in N\left(0,\;0.1\right)$, $v_{2R},\;v_{2I}\in N\left(0,\;20\right)$, P(c(k)=1)=0.06 and P(c(k)=0)=0.94. Figure 5 and Figure 6 present the performance of the RMCKRSL under a different kernel width σ and risk-sensitive parameter λ, respectively. One can see that both the kernel width σ and risk-sensitive parameter λ play an important role in the performance of the RMCKRSL. It is challenging to choose the optimal σ and λ because it is dependent on the statistical characteristic of the noise, which is unknown in the practical case. Thus, it is suggested that the parameters are chosen by experimentation.

### 4.2. Example 2

In this example, the superiority of the RMCKRSL is demonstrated by the prediction of a nonlinear system, where $s\left(t\right)=u_{0}\left[s_{1}\left(t\right)+js_{2}\left(t\right)\right]$, $s_{1}\left(t\right)$ is a Mackey-Glass chaotic time series described as follows [15]:

(34) $$\frac{ds_{1}\left(t\right)}{dt}=-0.1s_{1}\left(t\right)+\frac{0.2s_{1}\left(t-30\right)}{1+s_{1}{\left(t-30\right)}^{10}}\;$$

**Figure 7:**
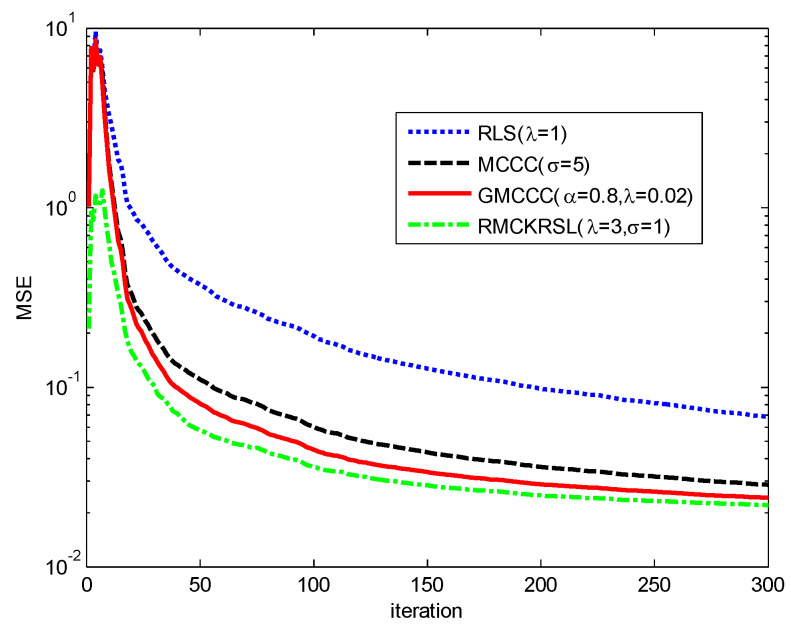
Convergence curves of different algorithms.

$s_{2}\left(t\right)$ is the reverse of $s_{1}\left(t\right)$, and $u_{0}$ is a complex valued number whose real and imaginary parts are randomly generated and obey a uniform distribution over the interval [0, 1]. s(t) is discretized by sampling with an interval of six seconds, and affected by the contaminated Gaussian noise $v\left(k\right)=\left(1-c\left(k\right)\right)v_{1}\left(k\right)+c\left(k\right)v_{2}\left(k\right)$, where $v_{1}\left(k\right)=v_{1R}\left(k\right)+jv_{1I}\left(k\right)$, with $v_{1R},\;v_{1I}\in$N(0, 0.1), $v_{2}\left(k\right)=v_{2R}\left(k\right)+jv_{2I}\left(k\right)$ with $v_{2R},\;v_{2I}\in N\left(0,\;20\right)$, P(c(k)=1)=0.06, P(c(k)=0)=0.94. s(k) is predicted by **x**(*k*) = [*s*(*k* − 1) *s*(*k* − 2) ⋯ *s*(*k* − 6)] and the performance is measured by the mean square error (MSE) with $\mathrm{MSE}\left(k\right)=\frac{1}{N-6}\sum_{l=7}^{N}\left({\left|s\left(l\right)-w^{H}\left(k\right)x\left(l\right)\right|}^{2}\right)$. The convergence curves of different algorithms on the basis of MSE are compared in Figure 7. One may observe that the RMCKRSL has a faster convergence rate and better filter accuracy than other algorithms. In addition, the RLS behaves the worst since the minimum square error criterion is not robust to the impulse noise.

## 5. Conclusions

As a nonlinear similarity measure defined in kernel space, kernel risk-sensitive loss (KRSL) shows a superior performance in adaptive filter. However, there is no report about the recursive KRSL algorithm. Thus, in this paper we focused on the complex domain adaptive filter and proposed a recursive minimum complex KRSL (RMCKRSL) algorithm. Compared with the MCCC, GMCCC and traditional RLS algorithms, the proposed algorithm offers both a faster convergence rate and higher accuracy. Moreover, we derived the theoretical value of the EMSE, and demonstrated its correctness by simulations.
